# The maximum standardized uptake value of ^18^ F-FDG PET scan to determine prognosis of hormone-receptor positive metastatic breast cancer

**DOI:** 10.1186/1471-2407-13-42

**Published:** 2013-01-31

**Authors:** Jian Zhang, Zhen Jia, Joseph Ragaz, Ying-Jian Zhang, Min Zhou, Yong-Ping Zhang, Gang Li, Bi-Yun Wang, Zhong-Hua Wang, Xi-Chun Hu

**Affiliations:** 1 Department of Medical Oncology, Department of Oncology, Fudan University Shanghai Cancer Center; Shanghai Medical College, Fudan University, Shanghai, China; 2 Faculty of Medicine, School of Population and Public Health, University of British Columbia, Vancouver, BC, Canada; 3 Department of Nuclear Medicine, Department of Oncology, Fudan University Shanghai Cancer Center; Shanghai Medical College, Fudan University, Shanghai, China; 4 Department of Medical Oncology, Fudan University Shanghai Cancer Center Minhang Branch, Fudan University, Shanghai, China

**Keywords:** Metastatic breast cancer, Luminal subtype, PET/CT, SUVmax, Prognosis

## Abstract

**Background:**

Whether PET scan maximum standard uptake value (SUVmax) could differentiate luminal A from luminal B and help predict the survival of metastatic breast cancer (MBC) patients with luminal subtype is still unknown and need to be investigated.

**Methods:**

305 MBC patients with luminal subtypes were screened with PET/CT. Eligible patients were prospectively followed up.

**Results:**

In total, 134 patients were eligible for this study. SUVmax was significantly related to the number of metastatic sites and presence of visceral metastasis on univariate analysis. SUVmax could not effectively differentiate patients with luminal A from luminal B subtype. Although luminal subtype at diagnosis could predict the relapse-free interval, it could not predict progression-free survival (PFS) or overall survival (OS) after developing relapse. In contrast, SUVmax was predictive of both PFS and OS and this effect was maintained in multivariate COX regression model.

**Conclusions:**

SUVmax of MBC did not correlate with molecular subtypes of primary tumor. While molecular subtype may be a valuable prognostic factor at primary diagnosis of breast cancer, the SUVmax, rather than molecular subtype, does have a potential to predict independently in multivariate analysis for the PFS and OS in patients with metastatic disease of luminal subtype.

## Background

Breast cancer is the most common female cancer. It affects almost 1.4 million women worldwide and about 459,000 patients die due to this disease every year [1]. Approximately 6% of women with breast cancer have metastatic disease at the time of diagnosis and about 20% of patients initially diagnosed with localized disease will develop metastatic breast cancer (MBC) [2]. Despite significant improvements in the treatment of MBC during the last decade, it remains an incurable disease, with a median life expectancy of 18–30 months [3].

Hormone receptors (HR), estrogen receptor (ER) and progesterone receptor (PgR), play important roles in breast cancer development, progression and response to therapy. The traditional classification of breast cancers into HR-positive and -negative groups helps to guide patient management. However, despite appropriate endocrine therapy, some HR-positive tumors recur and/or become metastatic. Microarray gene expression analysis (cDNA) has identified two biologically distinct HR-positive subtypes of breast cancer with significant differences in patient outcome: luminal A and luminal B [4]. However, cDNA analysis is too complex and costly and thus not routinely performed to identify breast cancer subtypes. A clinically relevant subtype classification can be obtained by immunohistochemical (IHC) analysis of the tumor expression of ER, PgR, HER2 or Ki67 [5]. IHC could also classify two categories of luminal subtypes: luminal A (ER and/or PgR-positive, HER2-negative), and luminal B (ER and/or PgR-positive, HER2-positive) [5]. However, compared to cDNA array, the IHC testing does not identify all the luminal B tumors because only 30% to 50% are HER2-positive on IHC. Thus, many luminal B tumors on cDNA array would be classified as luminal A on IHC. In 2009, Cheang et al. modified the IHC definition and found that Ki67 could distinguish on IHC the luminal A versus B subtype more accurately, with the Ki67 index cut point of 13.25% [6]. The luminal A subtype was then defined as HR-positive, HER2-negative breast cancer with Ki67 index < 14%, while luminal B subtype was defined as also HR-positive, but either HER2-positive, or HER2-negative with Ki67 index ≥ 14%. Compared with luminal A tumors, luminal B tumors have thus higher proliferation and poorer outcomes despite being clinically HR-positive. Consequently, the major biological distinction between luminal A and B is the proliferation signature, which includes genes such as CCNB1, MKI67, and MYBL2, with higher expression in luminal B than in luminal A tumors and may be important to breast cancer biology and prognosis [7,8].

Positron emission tomography (PET), using the radiolabeled glucose analog ^18^ F-fluorodeoxyglucose (^18^ F-FDG), can detect enhanced glycolysis of cancer cells and has proven valuable in diagnosing, staging, detecting recurrences, and assessing response to therapy in a multitude of malignant disorders [9]. Since ^18^ F-FDG uptake in cancer usually indicates the degree of tumor proliferation and metabolism, it was felt important to evaluate whether PET could be used as a noninvasive diagnostic modality to differentiate luminal A from luminal B tumors and hence predicting their behavior and prognosis. The standardized uptake value (SUV) is a semi-quantitative simplified measurement of the tissue FDG accumulation rate, and studies of the head and neck, lung, esophageal, endometrial, cervical and renal cell cancer have explored the prognostic significance of the maximum standardized uptake value (SUVmax) [10-16]. However, the role of the Baseline SUVmax as a prognostic factor for treatment naïve MBC patients of luminal subtype has not yet been evaluated so far.

The main objective of this study was to determine whether Baseline SUVmax in MBC patients correlates with validated prognostic markers and their luminal subtypes, and to establish whether the Baseline SUVmax could be used as a noninvasive indicator to differentiate luminal A from luminal B subtypes. In addition, we also prospectively investigated the impact of Baseline SUVmax on the survival of MBC patients with luminal subtype.

## Methods

### Study design and patient population

**Figure 1:**
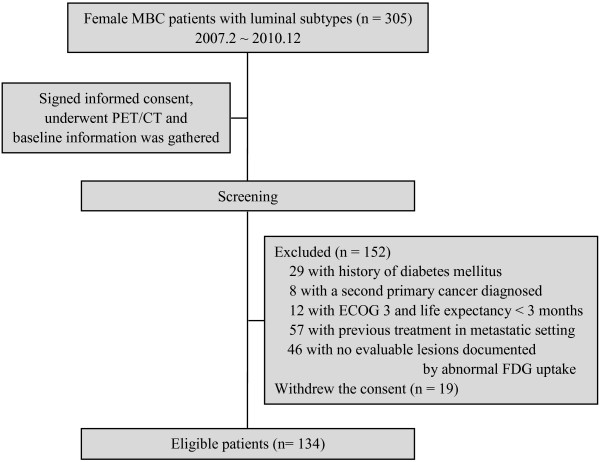
Patient screening and inclusion diagram.

Between February 2007 until December 2010, a total of 305 MBC patients with luminal subtypes (HR-positive) signed consent for this study and underwent PET/CT examinations. Baseline information collected, including PET/CT results, was then used to evaluate whether the individual was eligible for the study according to inclusion and exclusion criteria (Figure 1). Eligible patients were prospectively followed up at two-month intervals in Fudan University Shanghai Cancer Center (FUSCC), Shanghai, China. This study was approved by the FUSCC institutional ethical review board.

Originally we defined the luminal subtypes as defined by Carey et al. [5]. After 2009, we changed the diagnostic criteria and reviewed all the paraffin sections before. The new criteria were described according to ER, PgR, HER2 and Ki67 status [6]. We defined HR-positive, HER2-negative and Ki67 index <14% as luminal A, HR-positive and HER2-positive (or HER2-negative with Ki67 index ≥14%) as luminal B. Her-2/Neu status was defined positive when over-expressed with 3 plus staining in IHC or amplified with a ratio > 2.2 by fluorescence in situ hybridization (FISH). Ki67 was visually scored for percentage of tumor cell nuclei with positive immunostaining above the background level by two pathologists.

Criteria for inclusion were as follows: female gender, 18 to70 years of age, histologically confirmed breast cancer with luminal subtypes, eastern cooperative oncology group (ECOG) performance status of 0 to 2, life expectancy of > 3 months, written informed consent for the study participation, adequate bone marrow reserve, adequate liver and renal function, with no systemic or locoregional therapy in the metastatic setting, and at least one evaluable metastatic lesion with abnormal FDG uptake.

Exclusion criteria included: uncontrolled brain metastasis, pregnancy or breast-feeding, history of diabetes mellitus, diagnosis of second primary malignancy, and active or uncontrolled infection.

### ^18^ F-FDG PET, image analysis and information collection

^18^ F-FDG was produced automatically by cyclotron (Siemens CTI RDS Eclips ST) using Explora FDG4™ module at our single institution. PET/CT was performed using a PET/CT system (Siemens biograph 16HR). All patients were instructed to fast for at least 6 hours before PET imaging. At the time of the tracer injection, patients should have had a blood glucose level of less than 7.8 mmol/L. Before and after injection, patients were kept lying comfortably in a quiet, dimly lit room. There was no significant difference in blood glucose levels measured at the time of the pre- and post-^18^ F-FDG studies. Image acquisition was started 1 h ± 10 min after intravenous administration of FDG (7.4 MBq/kg body weight).

For the semi-quantitative analysis, a volume of interest (VOI) was drawn with a multimodality computer platform (Siemens) for each lesion with the largest uptake according to size and intensity. Tumor size had to be a minimum of 1 cm to minimize partial volume averaging effects in FDG-PET interpretation. Interpretation of the PET/CT images was based on assessment of the focal FDG uptake and a quantitative evaluation by calculating the SUVmax for each lesion instead of using the mean SUV of the lesion, which was more operator-dependent. Two nuclear medicine - CT diagnostic radiologists with at least 5 years of experience and unaware of the clinical information analyzed the data independently, and a third similarly qualified physician was asked for opinion in cases of discordance. The lesions with positive ^18^ F-FDG uptake were biopsied (n = 55, including 27 fine needle aspirations and 28 core biopsies), or assessed by further imaging and clinical follow-up (n = 79) to establish malignant characteristics.

Baseline information of the cohort including SUVmax and molecular subtypes was collected. For patients who had multiple metastatic sites, the single lesion with the highest SUVmax was used for calculation. All the information of molecular classification was obtained from the initial tumor sample from the primary surgery, with evaluation of patients’ tumor status performed using Response Evaluation Criteria in Solid Tumors (RECIST) v1.0 criteria. All patients were followed up at two-month intervals, and the data were collected and updated until February 25, 2012. Relapse-free interval (RFI) was defined as the interval between primary tumor and recurrence. Progression-free survival (PFS) was defined as the length of time from the date of the informed consent to disease progression or death from any cause. Overall survival (OS)_1_ was defined as the interval between the date of breast surgery and the date of death from any cause. OS_2_ was defined as the time from the date of the informed consent until the date of death from any cause.

### Statistical methodology

We present summary statistics for SUVmax as medians and interquartile ranges (IQRs), because data were not normally distributed (data not shown). The impact of different clinical parameters including luminal types on Baseline SUVmax was evaluated by Mann–Whitney U test (between 2 groups) or Kruskal-Wallis test ( ≥ 3 groups). Receiver operator characteristic (ROC) curves were used to identify potential SUV cutoffs predictive of different luminal subtype. An area under the curve of 1.0 would indicate a perfect test, whereas 0.5 would represent a noninformative test. Kaplan-Meier method was accessed for survival analysis. Prognostic variables identified by univariate analysis, with P < 0.1, were analyzed in the multivariate Cox model. All reported p-values were two-sided. Statistical significance levels were set at P < 0.05. Statistical analyses were performed with SPSS 16.0 (SPSS, Chicago, IL).

## Results

### Patient and tumor characteristics

Overall, 305 MBC patients with luminal subtypes signed informed consent documents and underwent screening consecutively, of whom 134 were eligible for this study and included into the final analysis (median age, 52 years; range, 28–74 years) (Figure 1). The median time from diagnosis of primary disease to MBC diagnosis was 32.1 months (range, 0.5–245.9 months), and most patients (64.2%) relapsed after 2 years. Out of all eligible patients, 75 (56.0%) were luminal A type, 59 (44.0%) were luminal B type. Visceral metastases were present in 70 patients (52.2%) and non-visceral metastases included only lymph node involved (13.4%), only bone involved (14.9%), only skin and soft tissue involved (3.7%), and mixed (20.1%). Before the PET/CT procedure, 2 patients (1.5%) did not receive any systemic treatment, 4 (3.0%) received adjuvant or neoadjuvant (adj/neo) chemotherapy only, 6 (4.5%) received adj/neo hormonal therapy only, 101 (75.4%) received adj/neo chemotherapy plus hormonal therapy, and 21 (15.7%) received regimens including targeting agents in the adj/neo setting. Other baseline characteristics are provided in Table 1. The median follow-up time of this cohort after inclusion was 26.6 months (range, 14.17–51.2 months).

**Table 1:** Patient characteristics and SUVmax comparisons between or among groups

**Characteristics**	**No. of patients (n=134)**	**Baseline SUVmax**		
		**Median**	**IQR**	*P*value*
Age				
≤ 50	63 (47.0%)	6.60	5.10–9.10	0.616
> 50	71 (53.0%)	6.85	4.80–9.80	
Menstruation status				
Pre-menopausal	53 (39.6%)	6.60	5.10–9.00	0.685
Post-menopausal	81 (60.4%)	6.85	4.85–9.75	
Histology				
IDC	124 (92.5%)	6.95	5.03–9.66	0.131
ILC	6 (4.5%)	6.33	5.15–9.90	
Others	4 (3.0%)	4.65	3.10–5.68	
Luminal subtype				
Luminal A	75 (56.0%)	6.75	5.10–9.20	0.744
Luminal B	59 (44.0%)	7.00	4.80–10.00	
Adjuvant/neoadjuvant therapy				
Only CT (± RT)	4 (3.0%)	6.68	5.76–9.05	0.887
Only HT (± RT)	6 (4.5%)	6.15	3.74–9.85	
CT + HT (± RT)	101 (75.4%)	6.75	5.15–9.25	
Therapy with TT	21 (15.7%)	7.00	4.75–10.25	
No	2 (1.5%)	5.50	4.50–6.50	
Relapse-free interval				
≤ 2 years	48 (35.8%)	6.25	4.81–9.20	0.592
> 2 years	86 (64.2%)	7.00	5.18–9.59	
No. of metastatic sites				
1	53 (39.6%)	6.10	4.60–9.08	0.002
2	37 (27.6%)	6.25	4.70–8.15	
≥ 3	44 (32.8%)	8.40	6.21–10.73	
Visceral metastasis				
Yes	64 (47.8%)	7.60	5.53–10.28	0.009
No	70 (52.2%)	6.32	4.80–7.73	
Only bone	20 (14.9%)	4.85	3.58–7.10	0.063
Others				
Only lymph node	18 (13.4%)	6.37	5.20–7.78	
Only skin & soft tissue	5 (3.7%)	10.1	4.45–10.30	
Mixed	27 (20.1%)	6.70	5.10–7.70	

SUVmax: the maximum standardized uptake value; No.: number; IQR: interquartile range; IDC: invasive ductal carcinoma; ILC: invasive lobular carcinoma; CT: chemotherapy; RT: radiotherapy; HT: hormonal therapy; TT: target therapy.

* Mann–Whitney U test (between 2 groups) or Kruskal-Wallis test (≥3 groups).

### Factors associated with baseline SUVmax

The current study evaluated the influence of age, menstruation status, tumor histology, luminal subtype, type of neo/adjuvant therapy, RFI, number of metastatic sites, and visceral metastasis on Baseline SUVmax with Mann–Whitney U test or Kruskal-Wallis test. If patients had multiple metastatic lesions, the maximum one of SUVmax values of these lesions was selected for statistical analysis. The results showed that SUVmax was significantly higher only in patients with more metastatic sites (P = 0.002) or with presence of visceral metastasis (P = 0.009). In patients without visceral metastases, SUVmax of patients with bone involved only had a trend to be lower than the others (P = 0.063) (Table 1).

### Evaluation of baseline SUVmax to differentiate luminal subtypes

**Figure 2:**
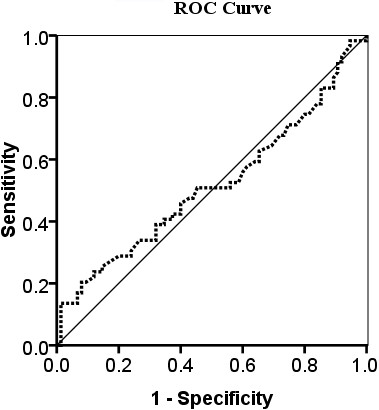
**The receiver operator characteristic (ROC) curve for SUVmax in the differential diagnosis of luminal A subtype from luminal B subtype.** The curve describes the association between sensitivity and specificity at different thresholds. The area under the curve (AUC) was 0.516.

The ROC curve was obtained by plotting a graph, in which the vertical axis showed sensitivity and the horizontal axis showed the false-positive rate. The area under ROC curve was 0.516 (SE 0.052) (SE is the standard error of the area estimate), which indicated that the Baseline SUVmax in the metastatic setting did not effectively separate patients with luminal A subtype from those with luminal B subtype (Figure 2).

### Baseline SUVmax and luminal subtypes as prognostic variables

**Figure 3:**
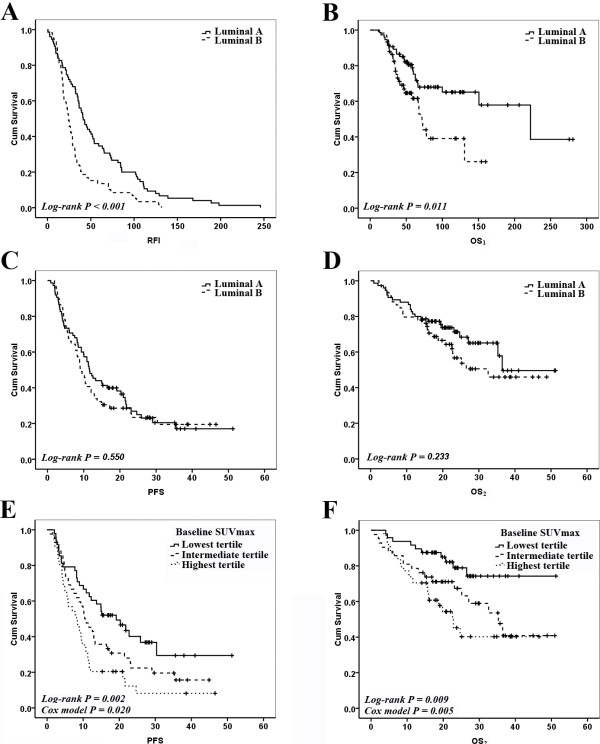
**Luminal subtypes and Baseline SUVmax as prognostic variables in survival curves. (A)** Relapse-free interval (RFI) curves according to Luminal types. **(B)** Overall survival 1 (OS_1_) curves according to Luminal types. **(C)** Progression-free survival (PFS) curves according to Luminal types. **(D)** Overall survival 2 (OS_2_) curves according to Luminal types. **(E)** PFS curves according to Baseline SUVmax tertiles. **(F)** OS_2_ curves according to Baseline SUVmax tertiles.

All patients in the study experienced disease relapse and developed metastases after primary breast surgery. Univariate analysis showed that luminal subtype was significantly associated with RFI (P < 0.001) and OS_1_ (P = 0.011), but not with PFS (P = 0.550) or OS_2_ (P = 0.233) (Table 2 and Figure 3A-D). Age, menstruation status, and tumor histology had no significant effect on PFS and OS_2_.

**Table 2:** **Univariate analysis of prognostic factors affecting RFI, OS**_**1**_**, PFS and OS**_**2**_

**Factors**	**Median RFI (months)**	*P*value*	**Median OS**_**1**_**(months)**	*P*value*	**Median PFS (months)**	*P*value*	**Median OS**_**2**_**(months)**	*P*value*
Age								
≤ 50	NA†		NA†		10.0	0.868	36.5	0.966
> 50					10.9		NR	
Menstruation status								
Pre-menopausal	NA†		NA†		9.5	0.985	36.5	0.565
Post-menopausal					11.3		NR	
Histology								
IDC	31.5	0.550	130.8	0.454	10.4	0.513	36.5	0.560
ILC	36.4		NR		4.4		NR	
Others	39.1		72.8		17.2		32.6	
Luminal subtype								
Luminal A	41.2	<0.001	222.1	0.011	11.6	0.550	36.5	0.233
Luminal B	24.2		71.7		9.4		32.6	
Adjuvant/neoadjuvant therapy								
Only CT (± RT)	17.7	0.138	NR	0.927	5.1	0.463	NR	0.639
Only HT (± RT)	17.2		NR		23.2		NR	
CT + HT (± RT)	36.1		NR		11.3		NR	
Therapy with TT	28.9		NR		8.4		NR	
No	28.9		NR		13.7		NR	
Relapse-free interval								
≤ 2 years	NA		NA		8.2	0.003	22.8	0.017
> 2 years					12.9		NR	
No. of metastatic sites								
1	NA		NA		13.0	0.002	NR	0.032
2					16.5		32.6	
≥ 3					8.4		25.1	
Visceral metastasis								
Yes	NA		NA		9.0	0.035	36.5	0.393
No					13.3		NR	
First-line therapy after PET/CT								
HT	NA		NA		21.0	0.037	NR	0.019
CT (± TT)					9.5		32.6	
Baseline SUVmax								
≤ 5.60 (Lowest tertile)	NA		NA		19.2	0.002	NR	0.009
5.60 ~ 8.70 (Intermediate tertile)					10.4		35.3	
> 8.70 (Highest tertile)					8.2		22.6	

RFI: relapse-free interval; OS: overall survival; PFS: progression-free survival; IDC: invasive ductal carcinoma; ILC: invasive lobular carcinoma; CT: chemotherapy; RT: radiotherapy; HT: hormonal therapy; TT: target therapy; No.: number; SUVmax: the maximum standardized uptake value; NA: not applicable; NR: not reached.

* Log-rank test.

† The data of age and menstruation status were collected after diagnosis of relapse and informed consent obtained. These data were different from those at diagnosis of primary breast cancer.

The univariate analysis also indicated that RFI ≤ 2 years (P = 0.003 and P = 0.017, respectively), more metastatic sites (P = 0.002 and P = 0.032, respectively), presence of visceral metastasis (P = 0.035 and P = 0.393, respectively), chemotherapy as the first-line therapy after PET/CT (P = 0.037 and P=0.019, respectively) and higher Baseline SUVmax (P = 0.002 and P = 0.009, respectively; Figure 3E-F) were significantly associated with shorter PFS and OS_2_ (Table 2). Here, the patients with different SUVmax were classified into three groups based on tertiles of SUVmax. Tertiles (as opposed to quartiles, quintiles, etc.) were chosen to balance the flexibility gained by adding more groups with the need to keep group sizes sufficiently large for subgroup analyses.

Cox regression analysis showed that Baseline SUVmax, RFI, and number of metastatic sites were three independent prognostic factors for PFS, while the significant predictors of OS_2_ in the regression model were Baseline SUVmax and RFI. Hazard ratios (HRs) for these factors are reported in Table 3. The significant prognostic effect of SUVmax on PFS and OS was maintained after correcting for tumor phenotype and variables with P < 0.1 on univariate analysis. By using the tertile with the lowest SUVmax as the reference group, patients in the highest tertile of SUVmax had the shortest PFS (HR = 2.06; 95% CI, 1.23-3.45) and OS (HR = 3.54; 95% CI, 1.66-7.55).

**Table 3:** Cox regression* results of PFS and OS_**2**_

***Cox regression results of PFS***			
Independent prognostic factors	Hazard ratio (HR)	95% CI	*P* value
Relapse-free interval			
≤ 2 years	Ref		
> 2 years	0.51	0.34-0.76	0.001
No. of metastatic sites			
1	Ref		
2	1.00	0.59-1.68	0.993
≥ 3	1.90	1.18-3.08	0.008
Baseline SUVmax			
Lowest tertile	Ref		
Intermediate tertile	1.60	0.97-2.66	0.067
Highest tertile	2.06	1.23-3.45	0.006
*Cox regression results of OS*_*2*_			
Independent prognostic factors	Hazard ratio (HR)	95% CI	*P* value
Relapse-free interval			
≤ 2 years	Ref		
> 2 years	0.44	0.25-0.78	0.005
Baseline SUVmax			
Lowest tertile	Ref		
Intermediate tertile	2.44	1.12-5.32	0.025
Highest tertile	3.54	1.66-7.55	0.001

* The procedure was carried out with the method of “Forward: LR”.

PFS: progression-free survival; OS: overall survival; CI: confidence interval; No.: number; SUVmax: the maximum standardized uptake value; Ref: reference category.

## Discussion

Given the fact that human breast cancer depends on HR signaling in regards to response to endocrine therapies, breast cancers have traditionally been sub-classified into HR-positive (or “luminal”) and HR-negative diseases. As identified, even the HR-positive or luminal cancers comprise a spectrum of tumors with varying degrees of proliferation and levels of genetic aberrations. Thus, “luminal type” of HR positive tumors can be further divided into subclass A and B with luminal B being higher grade, having higher proliferation index and a poorer prognosis independent on hormonal therapy. Since a significant correlation between FDG uptake in breast cancer by PET scan and proliferation index has been observed [17], and the tumor proliferation, as defined by microarray-based gene signatures or Oncotype DX testing (the 21 gene assay, Genomic Health), has been shown to be one of the strongest predictors of outcome for patients with HR-positive disease [18], it was important to establish whether SUVmax as identified by PET scanning could noninvasively differentiate luminal A from luminal B tumors, and whether SUVmax could predict the outcome of MBC patients with luminal subtypes.

The evaluation of Baseline SUVmax of metastatic sites after relapse may provide important information about tumor proliferation and metabolism that could be of prognostic significance. In this regard, a study of the association of Baseline SUVmax with other well-established prognostic factors could be an important first step towards establishing the relevance of FDG-PET in the prognostic characterization of MBC. Our study found that Baseline SUVmax of MBC was significantly associated only with number of metastatic sites and presence of visceral metastasis. This finding could be the result of accelerated glucose metabolism and related increased metabolic activity of those more aggressive metastatic tumor phenotypes. However, the location of metastatic lesion could also influence the SUVmax. For example, bone metastasis of breast cancer is often osteoblastic and osteoblastic bone metastatic foci usually show low FDG uptake, regardless of biologic behavior of tumor cells. In patients with visceral metastases (± non-visceral metastases) of this study, almost all the SUVmax were obtained from visceral lesions. Only in patients without visceral metastases, the assessment of influence on SUVmax by metastatic locations such as bone, lymph node, skin or soft tissue might be important. However, we did not find any significant differences among these locations (P = 0.235), even between patients with bone involved only and the others (P = 0.063) in terms of median SUVmax.

Our further investigation using the ROC curve indicated that the Baseline SUVmax in the metastatic setting could not differentiate luminal A from luminal B subtypes effectively. This might be because the difference between luminal A and B tumors in proliferation and metabolism was not substantial enough to influence SUVmax. Therefore, the SUVmax cannot be used as a noninvasive indicator to differentiate luminal A from luminal B subtypes. However, it should be noted that the association between Baseline SUVmax and luminal subtypes may be confounded by the fact that relapsed or metastatic lesions may have a different HR or HER2 status from that of the primary tumor [19-24] and that core biopsies in our study were performed only in a small proportion of patients at the time of relapse. Thus, in order to clarify whether SUVmax will have a role in luminal subtypes differentiation, it may be necessary to conduct another large-sample study to correlate SUVmax with core biopsies of the PET scan tested metastatic lesions. However, from 28 patients with core biopsies in our study, only 14.3% had discordant molecular subtypes with the primary lesions (Additional file 1: Figure 1). When Baseline SUVmax was used to differentiate the luminal subtypes after relapse in these patients, the area under ROC curve was 0.551 (SE 0.122) and still indicated no help to differentiate luminal A from luminal B (Additional file 2: Figure 2).

Sorlie et al. demonstrated that breast cancer can be classified into 5 different subtypes according to cDNA molecular profiles, and that these molecular subtypes significantly influence patient’s prognosis [4]. Subsequently, the study of Munoz et al. [25] showed a significantly unfavorable prognosis for luminal B patients in comparison to those with luminal A subtype in terms of RFI and OS_1_. Our data confirmed these results. However, in our study, we did not find different luminal subtypes predicting the PFS or OS_2_ after relapse, a phenomenon which could also be result of transformation of the original HR or HER2 status. Another reason for this could be a suboptimal accuracy in the measurement of our luminal subtypes, with a sensitivity of 77% (95% CI, 0.64-0.87) and a specificity of 78% (95% CI, 0.68-0.87) [6]. Hence, it was necessary to explore new indicator to determine prognosis of the MBC patients with luminal subtypes, and PET scan was of particular attraction due to its non-invasive nature.

Our study is one of the first to show that the Baseline SUVmax of PET scan has significant association with prognosis and outcome of MBC patients with luminal subtypes in terms of PFS and OS_2_, with the multivariate COX regression analysis confirming the SUVmax an independent prognostic factor.

Although some studies have examined PET/CT imaging as a predictor of treatment response in the primary breast cancer lesion [26-33], significantly less is known about how baseline PET/CT imaging can be used as a prognostic tool by quantifying radiotracer accumulation in metastases. A very recently published study showed that only in patients with newly diagnosed MBC to bone was Baseline SUVmax tertile significantly associated with OS on both univariate analysis (HR = 3.13) and multivariate analysis (HR = 3.19) [34]. However, this study was a retrospective one and did not focus on the patients with luminal subtypes.

Our study was prospectively performed with important findings for luminal type breast cancer patients with newly diagnosed metastases. Baseline SUVmax was found significantly related to the number of metastatic sites and presence of visceral metastasis but could not effectively differentiate patients with luminal A from luminal B subtype. Most importantly, although luminal subtype diagnosed according to the initial tumor sample from the primary surgery could predict the RFI, it could not predict PFS or OS_2_ after developing relapse or metastases. In contrast, Baseline SUVmax as determined on PET scan was predictive of both PFS and OS. In multivariate analysis using COX regression model, the Baseline SUVmax, RFI, and number of metastatic sites were three independent prognostic factors for PFS. For OS, the significant predictors were only Baseline SUVmax and RFI.

SUV has many drawbacks as it is dependent on parameters such as the delay between injection and measurement, plasma glucose concentration, body weight, instrumental factors and partial volume effect (PVE) [35]. SUVmax is defined as the SUV derived from the single voxel showing the highest uptake within a defined region of interest (ROI) or VOI. In the absence of noise, this SUVmax is indeed the least affected by PVE and so is often considered the best measure of tumor uptake. However, in any real imaging situation, noise is always present, making SUVmax variable. Another drawback of SUVmax is that because it is derived from a single voxel, it may not be an adequate surrogate marker for true tumor biology and it can be heavily influenced by voxel size [36]. Use of the maximum pixel value in a tumor to characterize tumor uptake, however, does make the measurement independent of the observer. This is why, despite its sensitivity to noise and voxel size, the use of SUVmax is still popular.

Several limitations of our study should be addressed. Firstly, as not all MBC patients in our center underwent PET/CT imaging, a selection bias may have played a role in our patients not being representative of general population of MBC cases with luminal disease. Secondly, more than a half of lesions with positive ^18^ F-FDG uptake were not biopsied, and thus metastatic disease was diagnosed only with imaging and long-time clinical follow-up. Thirdly, not all HER2-positive luminal B patients received trastuzumab, which may partly influence applicability of our results to the HER2-positive cases who will have trastuzumab therapy. Lastly, we examined PET/CT imaging from only 1 time-point and, thus, are unable to comment on the predictive effect of PET/CT imaging with regard to treatment effect.

In spite of these limitations, our study remains the first to establish the role of PET scanning as a noninvasive outcome indicator of luminal A versus luminal B MBC subtypes.

## Conclusions

We conclude that while the Baseline SUVmax in our study of MBC did not correlate with molecular subtypes of primary tumor, the SUVmax, rather than molecular subtype, emerged as a potential surrogate marker for survival with metastatic disease. These data indicate a promise of PET scan use for prognostic assessment of patients with MBC in general, with future studies required to clarify the PET scan role in refining, as a non-invasive procedure, the significance and, ultimately, individualized therapeutic options for different molecular subtypes.

## Abbreviations

SUVmax: Maximum standard uptake value; MBC: Metastatic breast cancer; PFS: Progression-free survival; OS: Overall survival; HR: Hormone receptors; ER: Estrogen receptor; PgR: Progesterone receptor; IHC: Immunohistochemical; PET: Positron emission tomography; FDG: Fluorodeoxyglucose; ECOG: Eastern cooperative oncology group; RECIST: Response Evaluation Criteria in Solid Tumors; VOI: Volume of interest; RFI: Relapse-free interval; IQRs: Interquartile ranges; ROC: Receiver operator characteristic; HR: Hazard ratio; PVE: Partial volume effect; ROI: Region of interest.

## Competing interests

The authors declare that they have no competing interests.

## Authors’ contributions

Conceived and designed the study: XCH, YJZ. Performed the study: JZ, ZJ, MZ, YPZ. Analyzed the data: JZ, ZJ, JR, GL, BYW, ZHW. Wrote the paper: JZ, ZJ, JR, XCH. All authors have read and approved the final manuscript.

## Pre-publication history

The pre-publication history for this paper can be accessed here:

http://www.biomedcentral.com/1471-2407/13/42/prepub

## Supplementary Material

Additional file 1: Figure 1 Summary of molecular subtype differences between the primary and relapsed or metastatic lesion in 28 patients with core biopsies after recurrence. Click here for file

Additional file 2: Figure 2 The receiver operator characteristic (ROC) curve for SUVmax in the differential diagnosis of luminal A subtype from luminal B subtype in patients with core biopsies after recurrence (luminal A, 13; luminal B, 12). The curve describes the association between sensitivity and specificity at different thresholds. The area under the curve (AUC) was 0.551. Click here for file
