# Supplementary material for: The maximum standardized uptake value of 18 F-FDG PET scan to determine prognosis of hormone-receptor positive metastatic breast cancer
Source: BMC Cancer. 2013 Jan 31;13:42. doi: 10.1186/1471-2407-13-42 (PMC3583732; doi:10.1186/1471-2407-13-42)
Supplement: Additional file 1: Figure 1 — Summary of molecular subtype differences between the primary and relapsed or metastatic lesion in 28 patients with core biopsies after recurrence. [file 1471-2407-13-42-S1.doc]

28 breast cancer
with core biopsies after recurrence

Concordant with primary

24/28 (85.7%)

Luminal A 13/16 (81.3%)

Luminal B 11/12 (91.7%)

Discordant with primary

4/28 (14.3%)

Luminal A to

Luminal B 1

HER2-positive 1

Triple-negative 1

Luminal B to

Luminal A 0

HER2-positive 1

Triple-negative 0
