# Supplementary figures and images for: The maximum standardized uptake value of 18 F-FDG PET scan to determine prognosis of hormone-receptor positive metastatic breast cancer
Source: BMC Cancer. 2013 Jan 31;13:42. doi: 10.1186/1471-2407-13-42 (PMC3583732; doi:10.1186/1471-2407-13-42)

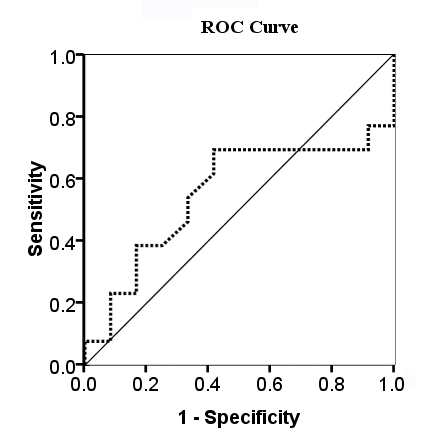

Supplement: Additional file 2: Figure 2 — The receiver operator characteristic (ROC) curve for SUVmax in the differential diagnosis of luminal A subtype from luminal B subtype in patients with core biopsies after recurrence (luminal A, 13; luminal B, 12). The curve describes the association between sensitivity and specificity at different thresholds. The area under the curve (AUC) was 0.551. [file 1471-2407-13-42-S2.tiff]
